# Surgical mistake causing an high recto-vaginal fistula. A case report with combined surgical and endoscopic approach: therapeutic considerations

**DOI:** 10.1186/1471-2482-13-S2-S7

**Published:** 2013-10-08

**Authors:** Michele Danzi, Fabozzi Massimiliano, Reggio Stefano, Pannullo Mario, Amato Bruno, Grimaldi Luciano

**Affiliations:** 1 Department of Specialized Surgery - Division of Gastrointestinal Surgery Rehabilitation of Election and Emergency. "Federico II" University, Naples - Italy; 2 Department of General Surgery. "U. Parini" Hospital, Aosta - Italy; 3 Department of General, Geriatric, Oncologic Surgery and Advanced Technologies. "Federico II" University, Naples - Italy

## Abstract

**Background:**

Rectovaginal fistulas (RVFs) have multiple causes, size and location on which the surgical treatment depends.

**Description:**

The Authors consider different approaches to RVFs and describe a clinical case of recurrent high RVF.

**Conclusions:**

Most RVFs can be successfully repaired, although many interventions may be necessary. A colostomy with delayed repair may improve RVFs outcome. Moreover, several authors indicate Mucosal Advancement Flap and Babcock-Bacon technique as the treatments of choice respectively for low and high RVFs (complex and recurrent) and emphasize the placement of endoscopic prothesis in cases of difficult healing of the anastomosis.

## Introduction

Rectovaginal Fistulas (RVFs) represent an arduous challenge even for the most expert surgeons can and a distressing situation for the women afflicted. Even if they only constitute about 5% of all the anorectal fistulas [1], numerous techniques have been suggested for their treatment, but unfortunately no one can assure a definitive result in the majority of cases.

The symptoms reported by majority of patients are unbearable for social, emotional, and sexual morbidity, and may sometimes be disabling. The RVF infact may cause the passage of gas and faecal material in the vagina giving out bad odor, a chronic vaginal or perineal suppuration and also the rectal syndrome with anal incontinence due to serious lesions of the anal sphincter. whose incidence in the women afflicted is very high (about 35% in the primiparous women and even 44% in the multiparous women) [2].

The anovaginal clinical examination and proctoscopy are essential for the diagnosis and definition of the anatomical and aetiological characteristics. If the RVF is of small dimension, it appears as a small depression on the vaginal mucosa, whose presence will be confirmed by a delicate probing of the passage. If the doubt persists, a tampon can be inserted in the vagina and methylene bleu injected into the rectum. thus the anal orifice is closed for 15-20 min and the tampon is examined: if it is not stained, a diagnosis of RVF is very improbable.

Other investigations (for the search of collateral passages and their related complications) include: fistulography, vaginography, barium enema, pelvic C.T. with contrast medium, anal manometry and when indicated electroneuromyography for the evaluation of the pudend nerve function [3].

## Clinical case

The clinical case we present concerns an incredible history of bilateral asyntomatic ovarien cysts, operated with several complications and followed by the appearance of a RVF.

R.J. is a 68 years old patient of, who is a carrier of HCV, with surgical antecedents of appendectomy (about 40 years ago) and hysterectomy (about 20 years ago). Hospitalized in the department of ginecology for two tumefactions of the ovaries, in April 2007 she had a bilateral annessectomy with some complications due to numerous and tenacious adhesive bridles, which were caused by previous surgical interventions. On this occasion. unfortunately it was not possible to avoid a lesion of the anterior wall of the Rectum, that forced the surgeon to perform the Hartman intervention. Her post-operation condition was normal. The patient was hospitalized and underwent surgery 2 months later to restore the intestinal continuity, (with the same difficulty found during the previous intervention, due to a complete freezing of the pelvis). Also on this occasion the ablation of visceral adherences unfortunately caused bladder and small intestine lesions which were immediately sutured. During hospitalization urine leekage from the abdominal drainage tube was found, thus a a re-intervention was needed. By opening the peritoneoum (which was almost nonexistent after several incisions), a left ureter lesion was individuated, so a termino-terminal anastomosis on probe with double J ends (stayed there for about 4 months) was made.

The urinary problem seemed to be resolved, but 6 days later the patient presented an output of dark and maleodorous material from the vagina, associated with a fever: a diagnosis of RVF was made. The patient was sent to us by Gynecologic Department to resolve this complication.

On clinical examination the patient was in a depressed state with asthenia, "abdominal facies". By using deep palpation, we observed a plethoric and less tractable abdomen, especially in the inferior quadrants, but peristalsis was present. Other clinical parameters were: temperature 37.8c c; cardiac frequency = 72 pm; blood pression = 140/ 80 mmHg.

The clinical and instrumental examinations showed a septic state due to the presence of a pelvic abscess identified by CT, associated with rectovaginal and enterocutaneous fistulas.

We positioned a venous central catheter (v.c.c.) and made a derivative loop colostomy on the right colon. At the same time appropriate antibiotic therapy was started. After this intervention the patient's general conditions seemed to be getting better, leucocytosis was reduced, renal function returned to normal except for a chronic infection of the urinary tract. The patient was without fever and the fistula seemed to be healed, so she was discharged in August, with the necessary medical treatment. During the check-up, a month after the discharge, the clinical examination seemed to confirm the healing of fistula, but the decision of restoring digestive continuity depended on the result of the pelvic C.T. and on the barium enema. Unfortunately these investigations confirmed the persistence of the fistula with the contrast liquid coming from the vaginal orifice, and the precence of a left pelvic subperitoneal abscess with diameter of 6 cm.

Another surgical intervention was needed to evacuate the abscess and to make a colectomy with tennino-terminal anastomosis according to Knight and Griffen. The post-operative course was normal and during the following clinical examination no vaginal o rectal side orifices were found.

Throughout the following months the patient was in good physical and psychological condition (previously numerous psychiatric consultations had been necessary to keep the patient from being depressed). The digestive function was good until the reappearance of the fistula at the fornix level, on the vaginal back wall, which is in communication with the rectum anterior wall at a distance of about 7 cm from the anus.

A surgical treatment was also needed in this occasion. After the positioning of a v.c.c., a resection of the previous colorectal anastomosis was made and a new coloanal anastomosis was performed according to Babcock-Bacon. After the mobilization of the colon, it was pulled out through the anus up to the healthy colic tract above the lesion Then it was dissected about 3 cm above the combed line with the excision of the fistula tract and the mucosectomy of the inside sphincter. The pulled out colon was fixed by a tape to a Foley n 18 catheter and to a glass rod to prevent a retraction, which were both removed on the 3^th ^day. The two stumps (colonic mucosa and anus derma) were anastomized on the 9^th ^day by local anesthesia. The post-operative course was complicated by bleeding between the distal colon and retracted rectal stump, and the attempt to treat it by *electrocaugulation *failed. As anastomosis reconstruction was not possible because of the excessive stump tension due to the previous resections, we decided to position a covered self-expanding endoanal prothesis. At the check-up 6 days later, the anal channel seemed to be healthy. The anterior part of the anastomosis was well healed over, but the posterior presented an opening of the anastomotic border with a residual bleeding due to the internal dislocation of the prothesis toward the top. Thus a second prothesis was placed below the first, to completely cover the anastomosis. The post-operative course was normal. Some days later the bleeding was stopped and the posterior part of the anastomosis was healing.

One year later the patient was in good general condition. The proctologic examination did not show anomalies and the prothesis was well positioned. The Methylene Blue injection through the anus did not show any fistulas. During vaginal inspection a residual scarred depression was found on the posterior wall but no fistulas were present. Thus confirming the healing of the wound. For these reasons we decided to close the colostomy. At the following check-up, 15 months later, the patient was free of disease.

## Discussion

Recurrent RVFs are a very difficult problem for surgeons. As suggested by the clinical case described there is not only one way of treating RVFs, but the approach depends on their etiology, anatomopathologic characteristics (for example the anal sphincter involvement) and previous attempts to repair them. RVFs are classified according to etiology, location and the dimensions.

They may also be classified as simple or complex (Table 1). Various techniques have been described to repair RVFs and their associated anatomical defects [4,5]. Their classification as simple or complex fistulas, other than reflecting the different anatomical characteristics, may also indicate the best treatment to use.

**Table 1:** RVFs Classification [24]

*AETIOLOGY:*
• *Congenital*
• *Acquired*
- *Traumatic (obstetric, post-operative, caused by rape or foreign bodies)*
*Infectious (perirectal abscesess. diverticulitis. tuberculosis, lymphogranuloma venereum, Bartholin gland abscesses)*
*Chronic linflommattny Bowel Diseases (Crohn is Disease and Ulcerative Colitis) Post-radiotherapy*
- *Neoplasms (primary. recurrent, metastatic)*
*Idiopatic*
*LOCATION:*
• *High (With the orifice fistulosus to the level of back fornix)*
• *Mid (between uterine cervix and the vaginal fork)*
• *Low (to the level of the vaginal fork): divided into oversphinteric and intrasphinteric*

*SIMPLE: Low or with diameter < 2.5 cm (traumatic or infectious)*
*COMPLEX: High with diameter > 2.5 cm (Inflammatory Bowel Diseses, post-radiorh., neoplasms).*

The surgical techniques could be divided according to the access used:

➣ **Perineal or low access **[6]: is recommended for simple RVFs and allows techniques with:

• Direct suture

- by perineum-vaginal access according to Musset [7];

- by trans-anal access according to the Mucosal Advancement Flap (MAF) [8];

Interventions of interposition of the Inside Rectum Mescle or according to Martius's technique and its variants [9];

➣ **Combined or mixed access: **is generally recommended for complex RVFs and allows:

• direct suture with or without epiploon interposition;

• rettoplasty according to Bricker [10];

• resection of the Rectum

- with trans-anal lowering of the colon (Babcock-Bacon's Pull Through resection or according to Parks) [11,12];

- with Knight-Griffen's anastomosis;

- Miles's abdomino-perineal amputation.

Lowry [13] reported a success rate of 88% in patients undergoing a primary repair of simple RVFs by MAF. The success percentage fell to 85% with the second repair and to 55% at the third attempt. This decreased success rate may be due to the presence of inflammation, tension, hematomas or underlying diseases, thus a different approach is recommended for fistulas known to have previously failed with MAF intervention. The MAF is also suitable for patients with distal RVFs; infact excellent results have been reported with this technique by some authors [14-16].

A simple colostomy presents a low rate of spontaneous healing (about 35.3%) [17]. Traumatic RVFs could spontaneously recover after the ablation of the foreign body, the drainage of the purulent collection and the protection colostomy above the lesion. In this case it is necessary to wait at least 3-6 months before surgical re-intervention Invasive laparotomy including re-anastomosis should never be performed and the rectal pull-through operation or abdominoperineal resection should only be reserved when other interventions fail [17]. Patients with sphincter damage should undergo sphincteroplasty using either fistulectomy or MAF as the first repair. Istead, MAF is not recommended for persistent complex fistulas. While Lowry suggests waiting for a period of 3-6 months before attempting a second repair, Hibbard affirms that waiting a period of 3 months is usually sufficient to resolve the acute state of inflammation and infection. RVFs repair of Lowry by the vaginal approach is not effective in the 90% of cases, so the trans-anal way according to Greenwald and Hoexter (Mucosal Advancement Flap) is preferred for the definitive resolution of the problem (this is obtained in 95% of cases) [8,18]. For these reasons the Greenwald's technique could represent the treatment of choice for its semplicity and for the best results. intact the complications rate (bleedings, infections, relapses) is very low (15%) in relationship to the other techniques proposed (Table 2). For high and complex RVFs the treatment of choice has not been fixed yet due to poor documentation is present in literature. Generally, the abdominal or abdominoperineal approach is preferable for these type of fistulas (neoplastic, attinic or in course of inflammatory bowel diseases RVFs) [19,20]. RVFs caused by inflammatory bowel diseases (IBD), have not possibility of spontaneous recovery, even if they are treated with antimetabolites therapies (Cyclosporine, 6-Mercaptopurine + Metronidazole, etc.) [21]. The same course have attinic or neoplastic fistulas. Simple RVFs have surely a better prognosis than high RVFs for which complex and aggressive techniques arc necessary. A pre-operative evaluation of the fistula's tract and its careful excision is very important for a successful treatment. It is also necessary to take multiple biopsies of the borders to exclude other possible underlying diseases. A functional evaluation of the anal sphincter using transanal ultrasonography, manometry and electromyography is also needed, in fact it may be damaged by the fistulous tract or during surgical interventions. In the case described techniques as manometry and endoscopy showed a normal sphincteric function and the absence of other lesions either in the pre- or in post-operative time. The MAF technique has not good results in complex or recidivant fistulas [22]. For these types of fistulas, the pull-through resection with coloanal anastomosis on 7-9^th ^day assures a complete excision of the fistolous tract. This operation, associated to an endoscopic positioning of the prothesis and a total parenteral nutrition for 14 days, allowes the recovery of the general patient's condition of the patient. In some cases, a preventive intestinal derivation for at least 3-6 months is necessary and is the only possible and definitive therapy.

**Table 2:** INDICATIONS OF SURGICAL TECHNIQUES

*AGE:*
• *Young women: less aggressive procedures with safeguard of vaginal integrity, when it is possible*
• *Elderly women: interventions for the drastic resolution of the problem with abdominoperineal approach;*
*ETIOLOGY:*
• *Post operative, traumatic and infectious RVFs*
- *MAF;*
- *recidivists: bowel resection with trans-anal anastomosis acc.to Babcock (or to Parks) + epiploon interposition;*
• *Post-radiotherapy RVFs*
- *colostomy + waiting for 3-6 months: if it's necessary, bowel resection with coloanal anastomosis or pull-throuh resection acc. To Babcock-Bacon;*
- *in absence of bleeding: bulbocavernosus muscle interposition am to Martius:*
- *in case of vulva sclerosis: inside rectum muscle interposition;*
- *when other attembt to repair fail -> definitive colostomy.*
• *IBD RVFs*
- *drainage qf perinal abscesses o trans-.sphinteric loop;*
- *if it's necessary: colostomy for 3-6 months;*
- *3 months of antimetabolites therapy:*
- *in more serious cases: bowel resections.*
• *Tumoral RVFs*
*low anterior resection of the rectum or amputation of Miles + back colpectomv;*

*LOCALIZATION:*
• *High RVFs requires interventions with combined approach;*
• *Low RVFs requires a perineal approach*
• *Mid RVFs require an approach depending on etiology and eventually associated lesions*

## Conclusions

This clinical case suggests us some important conclusions:

1. High RVFs therapy is not unique, but depends on different factors;

2. Surgery assure the complete excision of the fistulous tract;

3. The importance of a colostomy for the anastomosis protection, especially after reefing approach when the rectal mucosa is already damaged [23].

In our case the opening on the anastomosis and the fibrous retraction of the stumps with bleeding were treated infact with lowering the colic stump wasn't possible for its difficult isolation. We used an *autoexpandible covered endoprothesis *for the semplicity of positioning and for the therapeutic effect (hemostasis and prophilaxis of recidivant fistula); in fact, for a successful surgical repair, it is necessary to respect some principles such as handling and gentle dissection of tissues, a correct debridement and the colon mobilization to make tension-free anastomosis. Unfortunatlely no one is possible in our case.

We also want to confirm the MAF as the treatment of choice for the simple fistulas and not for the recidivant. For complex and recidivant fistulas, according to our experience, the methodic of the pull-through resection may have good results for the possibility of a radical excision. The reason for this aggressive approach is the lower rate of healing after a primary unsuccessful repair.

The endoscopic approach besides, in this specific case, was providential, infact it provided the protection of the rectal mucosa and in the same time the stop of the anastomotic collar bleeding. Thus we may propose the positioning of self-expandible covered endoprothesis when a protection of the rectal mucosa is needed. Simple RVFs repair by MAF has excellent results, while complex RVFs have to must be treated by more aggressive methods. In fact, once the first attempt to repair by derivative colostomy has failed, a difficult intervention such as the pull-through resection ace. to Babcock-Bacon must be managed by expert surgeons. Although in most cases repeated surgery may be necessary, the pull through resection is sure, radical and effective whereas other techniques have failed.

## List of abbreviations

RVF: recto-vaginal fistula; CT: computed tomography; HCV: hepatitis C virus; vcc: venous central catheter; MAF: mucosal advancement flap; IBD: inflammatory bowel disease.

## Competing interests

The authors declare that they have no competing interests.

## Authors' contributions

D.M.: conception and design, interpetration of data, given final approval of the version to be published.

F.M.: acquisition of data, drafting the manuscript, given final approval of the version to be published.

R.S.: acquisition of data, drafting the manuscript, given final approval of the version to be published.

P.M.: acquisition of data, drafting the manuscript, given final approval of the version to be published.

A.B.: critical revision, interpretation of data, given final approval of the version to be published

G.L.: conception and design, critical revision, given final approval of the version to be published.

## Authors' information

DM: Assistant Professor of Surgery at University Federico II of Naples.

FM: MD, PhD, in Surgery at U. Parini Hospital of Aosta.

RS: Resident in Surgery at University Federico II of Naples.

PM: Resident in Surgery at University Federico II of Naples.

AB: Associate Professor of Surgery at University "Federico II" of Naples.

GL: MD, Phd, in Surgery at University Federico II of Naples.

## Declarations

Funding for this supplement has come from personal funds.

This article has been published as part of *BMC Surgery *Volume 13 Supplement 2, 2013: Proceedings from the 26th National Congress of the Italian Society of Geriatric Surgery. The full contents of the supplement are available online at http://www.biomedcentral.com/bmcsurg/supplements/13/S2
